# Seasonal and Environmental Determinants of Maternal and Neonatal Vitamin D Status: A Cross-Sectional Observational Cohort Study in Urban Greece

**DOI:** 10.3390/healthcare13202568

**Published:** 2025-10-13

**Authors:** Artemisia Kokkinari, Maria Dagla, Kleanthi Gourounti, Antigoni Sarantaki, Giannoula Kirkou, Maria Iliadou, Evangelia Antoniou, Georgios Iatrakis

**Affiliations:** Department of Midwifery, School of Health & Care Sciences, University of West Attica, 12243 Athens, Greece; mariadagla@uniwa.gr (M.D.); kgourounti@uniwa.gr (K.G.); esarantaki@uniwa.gr (A.S.); ikirkou@uniwa.gr (G.K.); miliad@uniwa.gr (M.I.); lilanton@uniwa.gr (E.A.); giatrakis@uniwa.gr (G.I.)

**Keywords:** vitamin D, pregnancy, maternal–fetal health, UV index, PM2.5, air pollution, seasonality, urban environment, sunlight exposure, environmental epidemiology

## Abstract

**Background:** Cutaneous synthesis of vitamin D depends primarily on exposure to solar ultraviolet B (UVB) radiation. Nevertheless, populations in the Mediterranean region, including pregnant women, continue to experience high rates of hypovitaminosis D. Pregnancy is a particularly vulnerable period due to increased physiological demands and reduced outdoor activity. The aim of this study was to examine the seasonal and environmental determinants of maternal and neonatal vitamin D status in an urban Greek population. **Methods:** We conducted a cross-sectional observational study on 248 pregnant women and their neonates admitted for delivery at Tzaneio General Hospital of Piraeus between September 2019 and January 2022. Serum 25-hydroxyvitamin D [25(OH)D] concentrations were measured and temporally matched with environmental variables including UV index, sunshine hours, ambient temperature, and PM2.5 levels. **Results:** Both maternal and neonatal 25(OH)D levels exhibited marked seasonal variation, with levels peaking in late summer and declining sharply in winter. A significant positive correlation was observed between UV index and vitamin D concentrations (r = 0.45, *p* < 0.001), while elevated PM2.5 concentrations were inversely associated with vitamin D status. Despite supplementation, insufficiency persisted in most neonates, particularly during the low-UV season. This underlines the need for comprehensive prenatal care strategies, integrating both supplementation policies and individualized nutritional counseling, to better secure maternal and neonatal vitamin D adequacy. **Conclusions:** Seasonal and environmental factors, particularly solar radiation and particulate air pollution, have a decisive role in determining maternal and neonatal vitamin D status, even in regions with abundant sunlight. These findings emphasize the importance of adaptive prenatal care strategies that combine supplementation with dietary counseling and take into account seasonal variation and air quality. In addition, the study provides novelty by integrating maternal–neonatal vitamin D status with environmental exposure metrics such as UV and PM2.5.

## 1. Introduction

Vitamin D is a fat-soluble hormone critical not only for musculoskeletal health and calcium–phosphate balance but also for immune function and healthy fetal development [1,2]. Most of the body’s vitamin D, more than 80 percent, is naturally produced in the skin when it is exposed to ultraviolet B (UVB) radiation [3,4]. However, the efficacy of cutaneous vitamin D synthesis is influenced by several environmental and individual factors. However, its synthesis is modulated by geographic, individual, and environmental factors [5,6]. Climatic and air quality factors, particularly particulate matter (PM2.5) and nitrogen dioxide (NO_2_), have been shown to reduce UVB availability and influence vitamin D biosynthesis, especially in urban regions [7,8,9].

Paradoxically, vitamin D deficiency (VDD) is highly prevalent in countries situated at sun-abundant latitudes, particularly within the Mediterranean basin, a phenomenon commonly known as the “Mediterranean paradox” [10,11].

Despite abundant sunshine, studies consistently report high prevalence of insufficiency in southern Europe, including Greece, with up to 80% of adults presenting serum 25(OH)D levels below 30 ng/mL during winter, and substantial rates of insufficiency persisting even in summer [12]. In Southern European populations, studies have shown that up to 70% of pregnant women remain vitamin D insufficient throughout gestation, regardless of supplementation [13]. Similarly, high rates of deficiency have been documented among infants, adolescents, and reproductive-age women across Italy, Portugal, and Spain [14].

These findings challenge the assumption that geographic latitude or annual sunshine is sufficient to ensure vitamin D adequacy. They highlight the need to study how factors like solar angle, pollution, and seasonal cloud cover influence vitamin D levels, especially in vulnerable groups such as pregnant women.

Greece represents a particularly notable case within this paradox. Despite its favorable latitude (~37–41° N) and ample sunlight, national data show that many people, especially pregnant women and newborns, have low vitamin D levels. In urban Greek populations, more than 80% of pregnant women and up to 95% of newborns present serum 25(OH)D concentrations below sufficiency at birth [15]. Earlier and more recent studies alike confirm persistently high deficiency rates among mother–newborn pairs in Athens [16,17]. Furthermore, maternal vitamin D levels during pregnancy have been associated with key neonatal health outcomes in Greece, underscoring the public health importance of addressing VDD in this population [18].

Pregnancy constitutes a physiologically demanding state during which maternal vitamin D stores are critically important for both maternal skeletal health and fetal development. Physiological changes and reduced sun exposure during pregnancy may further compromise vitamin D status [19,20]. These vulnerabilities are exacerbated in urban areas, where air pollution such as PM2.5 and NO_2_ reduces dermal UVB-mediated vitamin D synthesis [4,8].

Recent studies highlight that pregnant women living in polluted, densely populated urban environments exhibit disproportionately high rates of hypovitaminosis D, even in regions with abundant sunlight [13,21]. Therefore, environmental quality must be considered a critical determinant in prenatal vitamin D sufficiency, particularly in climate-sensitive public health strategies. In this context, climate variability such as cloud cover, temperature inversions and pollution episodes can compound urban atmospheric effects and exacerbate seasonal reductions in UVB exposure. These phenomena are expected to intensify under future climate change scenarios, thereby heightening risks of VDD in pregnant populations. Dietary intake remains a modifiable factor, and during pregnancy nutritional counseling is a key preventive strategy.

Several mechanisms may account for the discordance between theoretical UVB exposure and biological vitamin D sufficiency. Seasonal variation in solar zenith angle leads to insufficient UVB during winter months, and at latitudes 37–41° N dermal synthesis becomes largely ineffective from October to March [22,23]. Estimates suggest that achieving sufficiency through sunlight alone would require prolonged daily exposure under ideal conditions, which are rarely met in urban environments [24]. Urban air pollution, particularly particulate matter (PM2.5), nitrogen dioxide (NO_2_) and ground-level ozone (O_3_), can reduce ground-level UVB by up to 30–60%, further limiting dermal synthesis [8,25,26,27,28].

Pollution can also affect vitamin D metabolism beyond blocking UVB. Toxins may induce oxidative stress and inflammation, disrupting vitamin D metabolism and receptor activity [4,29]. Endocrine-disrupting pollutants may impair placental transfer, reducing neonatal vitamin D even when maternal levels are adequate [18].

These multiple mechanisms show that UV availability alone is not a reliable indicator of vitamin D sufficiency in urban populations, highlighting the need for guidelines that integrate environmental conditions. At the same time, indoor lifestyles, limited sun exposure and poor adherence to supplementation can further worsen vitamin D insufficiency. Some researchers emphasize individual factors as primary determinants, while others stress the underestimated role of environmental conditions in urban areas [8,20].

Recent studies in urban climatology suggest that even within a single city, there can be significant variation in solar UVB exposure, air pollution and temperature due to urban structural and environmental variability [30]. In Athens, both satellite and ground data confirm notable variation in PM2.5 levels, temperature and urban heat island intensity [31,32]. Such neighborhood-specific differences may affect vitamin D synthesis, particularly in vulnerable groups such as pregnant women. These differences underline the importance of considering local rather than city-wide averages when assessing maternal health risks.

Despite increasing awareness, only a few studies have fully combined vitamin D measurements with environmental exposure data in real Mediterranean populations. Greece is a particularly relevant case, with marked seasonal UV variation and high urban pollution, especially in Athens [33,34]. This study aimed to investigate how seasonal and environmental factors influence maternal and neonatal vitamin D status in an urban Greek population by combining biomarker data with high-resolution environmental indicators (UV index, temperature, sunshine duration, PM2.5). To our knowledge, this is the first study in Greece to systematically link such environmental exposures with maternal–neonatal vitamin D outcomes. Understanding these interactions is essential for designing prenatal care strategies that combine supplementation with dietary guidance. Our findings are intended to inform climate-aware public health policies for pregnant populations in sun-rich but polluted regions. Therefore, the objective of the present study was to examine the prevalence of vitamin D deficiency among pregnant women and their newborns in an urban Greek population, and to investigate how seasonal and environmental factors—including UV index, temperature, sunshine duration, and air pollution—interact with maternal characteristics and supplementation practices to determine maternal–neonatal vitamin D status.

Based on these considerations, the present study set out to investigate the prevalence of VDD among pregnant women and their newborns in an urban Mediterranean setting. It further examined how seasonal and environmental factors, including UV index, temperature and air pollution, influence maternal and neonatal vitamin D levels. Finally, it explored the extent to which nutritional and lifestyle factors interact with environmental exposures to shape vitamin D status during pregnancy.

The novelty of this work lies in the integrated linkage of maternal–neonatal vitamin D status with seasonal UV metrics and air pollution (PM2.5) within the Greek urban context, rather than in the rediscovery of the Mediterranean paradox itself.

## 2. Materials and Methods

### 2.1. Study Design and Population

This was a cross-sectional observational cohort study with linked environmental exposures, conducted at Tzaneio General Hospital of Piraeus between September 2019 and January 2022. A total of 248 pregnant women were consecutively recruited at delivery together with their newborns. Eligible participants were either of Greek origin or had resided in Greece for at least ten years, ensuring long-term exposure to the Mediterranean climate. Inclusion criteria further required age ≥ 18 years, singleton live birth, and gestational age ≥ 37 weeks. Exclusion criteria comprised chronic kidney or liver disease, autoimmune, endocrine, or malabsorptive disorders, obesity, use of medications known to affect vitamin D metabolism (e.g., anticonvulsants, corticosteroids, antifungals, antituberculosis drugs), high-dose vitamin D supplementation (>800 IU/day), multiple gestations, stillbirth, or major fetal anomalies. The final study population consisted of 248 mother–newborn pairs. During the study period, 312 pregnant women were initially approached for participation. Of these, 64 were excluded due to ineligibility (multiple gestation, preterm delivery < 37 weeks, chronic disease, or use of medications affecting vitamin D metabolism). The final analytic cohort comprised 248 term singleton pregnancies without major complications. A completed STROBE checklist is provided as Supplementary Material (Table S1). Environmental exposure data (UV index, sunshine duration, ambient temperature and PM2.5 concentrations) corresponding to each mother’s gestational period were subsequently linked to individual-level biomarker data.

The primary outcomes were maternal and neonatal serum 25-hydroxyvitamin D [25(OH)D] concentrations, measured at delivery in mothers and from cord blood in newborns. Explanatory variables included seasonal and environmental indicators: daily UV index, sunshine duration, mean ambient temperature, and PM2.5 concentrations during pregnancy. Additional covariates were collected from maternal medical records and included age, body mass index, parity, smoking status, and vitamin D supplementation.

Maternal venous blood samples were obtained at delivery, and cord blood was collected immediately after birth. All blood samples were collected as part of routine pre-delivery laboratory testing by trained obstetric staff, without introducing additional procedures or risk to the mother or neonate. Environmental data were retrieved from the National Observatory of Athens and the Hellenic National Meteorological Service. Daily UV index, sunshine duration, mean ambient temperature and PM2.5 concentrations were extracted for the greater Athens area and matched to each participant’s gestational period.

The sample included only healthy term pregnancies (≥37 weeks) without known comorbidities or use of medications affecting vitamin D metabolism. Participants were either native Greeks or European residents living in Greece for more than 10 years. All deliveries occurred in an urban Mediterranean setting (latitude 37°56′ N), within the greater Athens-Piraeus metropolitan area.

Informed consent was obtained from all participants, and the study received ethical approval from the hospital’s scientific committee (Protocol No. 5844/29-3-2018). The study was conducted in accordance with the Declaration of Helsinki. Participation was voluntary, and mothers were informed that they could withdraw at any time without affecting their medical care. All personal data were anonymized and handled in compliance with institutional and national regulations.

### 2.2. Vitamin D Measurements

Blood samples were collected on the day of delivery. Maternal venous blood and neonatal umbilical cord blood were analyzed for serum 25-hydroxyvitamin D [25(OH)D] concentrations using the ARCHITECT 25-OH Vitamin D CMIA assay (Abbott Diagnostics, Sligo, Ireland), traceable to NIST Standard Reference Material 2972 (NIST SRM 2972) [35]. The assay range was 3.4–155.9 ng/mL.

Vitamin D status was defined according to the Endocrine Society guidelines [36], and participants were categorized into three clinically relevant subgroups:

Deficient: <20 ng/mL;
Insufficient: 20–30 ng/mL;
Sufficient: >30 ng/mL;
Severe deficiency: <12 ng/mL.

### 2.3. Seasonal Categorization

To assess the role of seasonality, data were divided into two climate-based groups, following Hellenic National Meteorological Service (HNMS) definitions:

Group A (Warm period): April 1–October 14;
Group B (Cold period): October 15–March 31.

These divisions align with UV radiation and sunshine seasonality in the Mediterranean climate.

### 2.4. Environmental and Climatic Data

For each month of sample collection (September 2019–January 2022), environmental variables for Athens/Piraeus were obtained from national and international providers, including the NASA POWER database, the National Observatory of Athens (NOA), the Hellenic National Meteorological Service (EMY), and the Ministry of Environment/OpenAQ platform. Variables included UV index, solar radiation, sunshine duration, ambient temperature, rainfall, and air pollution indicators (PM2.5, NO_2_, O_3_). Monthly means were aligned to the corresponding gestational months for each participant to enable correlation analysis. Environmental data were linked retrospectively to each gestational period (ecological linkage), while the overall study design remained a cross-sectional observational cohort.

Detailed provider URLs are provided in the Supplementary Methods.

### 2.5. Questionnaire and Participant Characteristics

Maternal demographic, lifestyle, and clinical information were obtained through structured questionnaires administered by trained research midwives in face-to-face interviews during the immediate postpartum hospitalization period. Consecutive recruitment was applied, including all eligible women admitted for delivery during the study period. To ensure accuracy, questionnaire responses were cross-checked with medical records whenever possible.

Information collected included age, parity, smoking status (non-smoker, current smoker, or quit during pregnancy), physical activity, daily sun exposure (hours/day, self-reported), socioeconomic status (self-reported as low/middle/high), and vitamin D supplementation (dose and duration). Clinical data such as pre-pregnancy BMI, gestational weight gain, and obstetric history were extracted from medical records.

Neonatal characteristics (gestational age, birth weight, length, and head circumference) were recorded immediately after birth by trained midwives using calibrated equipment. Birth weight was measured as the average of three consecutive measurements obtained within the first hour of life, in line with standardized procedures, to ensure reliability and consistency.

The questionnaire was designed by the study team after reviewing previous maternal–child health studies, ensuring consistency with variables commonly examined in Greek and European cohorts. While no formal pilot testing was conducted, its validity was enhanced by cross-checking self-reported responses with medical records whenever possible.

### 2.6. Statistical Analysis

Descriptive statistics were used to summarize maternal and neonatal vitamin D concentrations and participant characteristics. Continuous variables were expressed as means ± standard deviations (SD) when normally distributed, and as medians with interquartile ranges (IQR) when skewed, while categorical variables were summarized as frequencies and percentages. Normality was assessed using the Kolmogorov–Smirnov test. Maternal 25(OH)D concentrations were approximately normally distributed in both warm and cold seasons (mean ± SD: 24.22 ± 12.57 ng/mL vs. 16.96 ± 9.60 ng/mL), whereas neonatal 25(OH)D levels were right-skewed (median: 14.6 ng/mL, IQR: 10.2–19.8). Maternal age also followed a normal distribution, while BMI and environmental variables (UV index, PM2.5, sunshine hours) deviated from normality. For normally distributed variables, parametric tests were applied (independent samples *t*-test, one-way ANOVA, and Pearson correlation), while non-parametric equivalents (Mann–Whitney U test, Kruskal–Wallis test, Spearman correlation) were used for skewed variables. Associations between categorical variables, such as supplementation status and deficiency categories, were examined with Chi-square tests or Fisher’s exact test when expected cell counts were <5. Univariate analyses were first performed to assess crude associations between maternal vitamin D levels and demographic, behavioral, and environmental covariates, and variables significant at *p* < 0.10 were subsequently entered into multivariate linear regression models with maternal 25(OH)D as the dependent variable. Predictor variables included season (warm vs. cold), daily sun exposure, BMI, smoking status, parity, and supplementation use, with regression coefficients (β) and 95% confidence intervals (CI) reported. Logistic regression models were additionally constructed to identify predictors of maternal vitamin D deficiency (<20 ng/mL), and results were presented as odds ratios (OR) with 95% CI. All analyses were two-tailed with significance set at *p* < 0.05, and were performed using IBM SPSS Statistics version 26.0 (IBM Corp., Armonk, NY, USA) and R software (v4.2).

Multivariable regression results are reported with β coefficients (or odds ratios for logistic models), 95% confidence intervals, and adjusted R^2^. Full model outputs are provided in Supplementary Table S2. Analyses were exploratory, and no formal correction for multiple testing was applied; significance was set at α = 0.05, and results should be interpreted accordingly.

## 3. Results

### 3.1. Vitamin D Status by Season

In this cross-sectional cohort of 248 mother–infant pairs, a pronounced seasonal variation in serum 25(OH)D concentrations was observed in both pregnant women and neonates. During the cold season (Group B: mid-October to March), mean maternal 25(OH)D levels were 16.96 ± 9.6 ng/mL, while neonatal levels averaged 12.87 ± 8.2 ng/mL, both of which fell into the deficiency range (<20 ng/mL).

In contrast, during the warm season (Group A: April to mid-October), maternal vitamin D levels improved significantly to 24.22 ± 12.57 ng/mL, and neonatal levels reached 16.37 ± 8.55 ng/mL. Despite this seasonal improvement, maternal levels remained in the insufficiency range (20–30 ng/mL), and neonatal levels remained below 20 ng/mL, thus still categorized as deficient.

These seasonal trends are consistent with known UVB radiation patterns in southern Europe. Furthermore, ongoing urbanization and climate change exacerbate air pollution and atmospheric conditions, potentially intensifying UV attenuation and altering seasonal UVB patterns, which could worsen vitamin D synthesis in urban populations. At latitude 37°56′ N, the UV Index peaks between May and August (mean monthly UVI ≈ 9–10), but ambient environmental factors—including air pollution and behavioral sun-avoidance in pregnancy—may attenuate effective UVB exposure.

Notably, these findings align with Mediterranean climatic expectations, where solar UV availability drops significantly from October through March, due to both shorter photoperiod and atmospheric interference, such as haze and high PM2.5 concentrations.

Environmental variables for key months are summarized in (Table 1), highlighting the substantial decline in UV Index and sunshine duration during winter, alongside increased PM2.5 concentrations.

**Figure 1:**
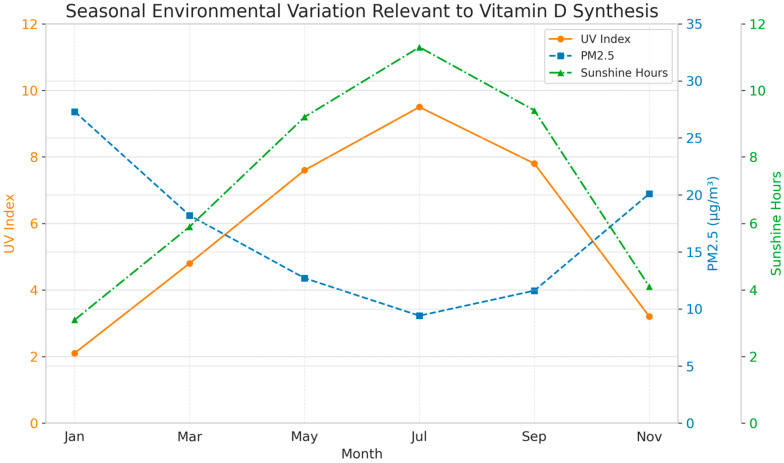
Climatic and Atmospheric Determinants of Maternal Vitamin D Status: A Seasonal Overview from a Southern European Urban Cohort.

A significant positive correlation was found between monthly mean UV index and average maternal 25(OH)D levels across the study period (r = 0.45, *p* < 0.001, Pearson correlation), supporting the observed seasonality. Figure 1 illustrates the seasonal dynamics of key environmental variables (UV Index, PM2.5 concentrations, sunshine hours, and temperature) across representative months in Southern Europe. Overlaid maternal 25(OH)D values indicate notable concordance with UV availability and sunshine duration, particularly during the warm season. This integrative visualization underscores how climate-linked environmental patterns—and particularly reduced UV exposure during winter months—may contribute to persistent maternal vitamin D insufficiency, even in traditionally high-irradiance regions.

Overall, the data reflect a persistent burden of suboptimal vitamin D status across both seasons, suggesting that natural UV exposure—even in high-sunlight environments—may not suffice to maintain adequate maternal–fetal vitamin D status without intervention (see Table 2). This underlines the importance of complementary strategies, including dietary intake and targeted prenatal counseling, to ensure sufficient vitamin D supply for both mother and infant throughout the year.

### 3.2. Prevalence of Vitamin D Deficiency

Despite the seasonal improvement observed in Group A, vitamin D deficiency remained highly prevalent year-round, particularly among neonates. In Group A (warm season), 75% of mothers and 92% of neonates had serum 25(OH)D levels < 30 ng/mL. These rates rose significantly in Group B (cold season), reaching 89% of mothers and 96% of neonates, despite moderate ambient sun exposure (Table 3).

Such prevalence rates, even in sun-abundant seasons, underscore the need for reevaluating current vitamin D guidelines and supplementation policies under climate-urban scenarios. These findings are particularly relevant for at-risk populations such as pregnant women, where insufficiency affects both maternal and fetal outcomes. Given the persistently high prevalence, even in sun-abundant seasons, routine prenatal care should incorporate not only supplementation but also nutritional counseling on vitamin D–rich foods, to mitigate risk factors beyond environmental limitations.

### 3.3. Maternal–Neonatal Vitamin D Correlation in an Environmental Context

A strong positive linear relationship was observed between maternal and neonatal serum 25(OH)D concentrations, with a Pearson correlation coefficient of r = 0.800 (*p* < 0.001), indicating that higher maternal levels strongly predict higher neonatal levels (Table 4, Figure 1).

While this correlation has been well-documented in previous obstetric studies, its relevance within the current environmental framework is noteworthy: both maternal and neonatal vitamin D levels were concurrently shaped by shared seasonal UV exposure and urban atmospheric conditions, particularly during periods of low insolation and elevated PM2.5.

These findings highlight the extent to which ambient environmental factors modulate not only individual but also dyadic (mother–infant) vitamin D dynamics, reinforcing the potential for climate-adapted antenatal supplementation strategies.

### 3.4. Supplementation and Environmental Exposure Interactions

Among the participants, 83 pregnant women reported taking prenatal vitamin D supplementation (400–800 IU/day), while 165 received no supplementation. Supplemented mothers exhibited significantly higher serum 25(OH)D levels compared to non-supplemented ones (26.92 ± 12.43 ng/mL vs. 16.92 ± 9.57 ng/mL, *p* < 0.001, independent samples *t*-test), although the majority remained within the insufficiency range.

Despite improved maternal levels, 73% of neonates born to supplemented mothers remained vitamin D deficient (<20 ng/mL), highlighting a disconnect between maternal intake and neonatal status (Table 5).

Seasonal stratification further revealed that neonatal deficiency remained high even among supplemented pregnancies during the cold season (Table 6).

This suggests that standard supplementation protocols may be insufficient to overcome environmental barriers, such as reduced ambient UVB exposure due to seasonal variation and urban air pollution.

Specifically, the majority of supplemented mothers delivered during the winter months, when UV Index values are lowest (≈2–3) and PM2.5 concentrations are highest, potentially compromising dermal synthesis of vitamin D despite oral intake.

As shown in Table 7, supplemented mothers had significantly higher mean serum 25(OH)D concentrations and lower rates of deficiency compared to non-supplemented women, although insufficiency remained common across both groups.

These findings stress the need for pollution-aware strategies and a climate-sensitive re-evaluation of prenatal dosing guidelines, particularly under environmental conditions of high atmospheric pollution and UV attenuation in urban Mediterranean settings.

This underscores the necessity to incorporate high-resolution environmental exposure data, such as real-time PM2.5 monitoring and UV dosimetry, into future prenatal care protocols to better tailor vitamin D supplementation under varying climatic and pollution scenarios. Equally, individualized prenatal care should emphasize adequate dietary sources of vitamin D and balanced maternal nutrition, given the limited transfer of supplementation benefits to neonatal stores under adverse environmental conditions.

### 3.5. Associations with Demographics and Environmental Exposure

In univariate analysis, maternal serum 25(OH)D concentrations were significantly associated with season (*p* < 0.001), daily sun exposure duration (*p* = 0.014), smoking status (*p* = 0.002), and parity (*p* = 0.004). Group differences were assessed using independent samples *t*-test or Mann–Whitney U test for continuous variables, and Chi-square test for categorical comparisons. No significant associations were observed with BMI (*p* = 0.722), socioeconomic status (*p* = 0.202), or physical activity (*p* = 0.508), and these variables were not retained for further analysis due to their limited environmental relevance (Table 8).

Sun exposure was significantly associated with higher maternal 25(OH)D levels, though its biological effectiveness may have been modulated by environmental UV attenuation and high particulate air pollution (PM2.5) during certain months. Indeed, certain participants with high sun exposure reported low 25(OH)D levels, particularly during winter months characterized by low ambient UVB availability and elevated PM2.5 concentrations.

To further investigate the independent contribution of the identified variables, a multivariate linear regression model was constructed (Table 9). Season and sun exposure remained significant predictors, while smoking retained borderline significance and parity lost statistical strength. A multivariate linear regression model was applied with maternal 25(OH)D as the dependent variable, and results are presented as standardized β coefficients with *p*-values. These findings suggest that public health policies addressing maternal VDD must be climate-responsive, integrating seasonal forecasts and urban pollution trends to inform dynamic supplementation guidelines and preventive strategies. Collectively, these results demonstrate that the statistical analyses extended beyond descriptive reporting, providing inferential evidence for the independent contribution of seasonality, environmental exposure, and maternal characteristics to vitamin D status. Overall, these results underscore the primacy of environmental conditions—particularly seasonal UVB availability—in shaping maternal vitamin D status, even when adjusting for behavioral and clinical factors.

## 4. Discussion

This study adds novelty by explicitly linking maternal–neonatal 25(OH)D concentrations with high-resolution seasonal UV and PM2.5 data in Greece, providing a context-specific environmental integration beyond the general concept of the Mediterranean paradox.

The analysis focused on the environmental and seasonal determinants of serum 25(OH)D concentrations in a maternal–neonatal cohort residing in urban Greece. Despite Athens’ location within a Mediterranean zone of high solar availability, the majority of both pregnant women and neonates demonstrated persistently insufficient or deficient vitamin D levels year-round. Mean maternal levels ranged from 16.96 ng/mL in winter to 24.22 ng/mL in summer (Table 2), while neonatal values remained consistently deficient across seasons. These patterns underline the importance of investigating how urban climatic conditions and atmospheric dynamics—particularly seasonal UVB attenuation and ambient air pollution—interact to constrain vitamin D synthesis in high-sunlight environments. Recent cohort studies have demonstrated that prenatal exposure to air pollution, particularly PM2.5, is associated with lower maternal 25(OH)D levels [37,38]. These findings complement our results by reinforcing the role of urban atmospheric exposures as key environmental determinants of vitamin D status in pregnant populations.

These findings support the concept of the “Mediterranean vitamin D paradox” [10,18], whereby populations in high-UV regions paradoxically exhibit high prevalence of hypovitaminosis D. In our sample, 75% of mothers and 92% of neonates in the warm season remained below sufficiency thresholds (Table 3), suggesting that environmental UV availability does not equate to biological sufficiency.

This paradox has been consistently documented across Southern Europe, where despite high annual sunshine, up to 80% of adults have been reported to present insufficiency during winter, with notable persistence into summer [12]. Similar patterns are observed in high-risk groups, with studies reporting that up to 70% of pregnant women remain insufficient during gestation, and deficiency is also prevalent among infants, adolescents, and reproductive-age women across Italy, Portugal, and Spain [13,14]. These findings underscore that geographic latitude and sunshine exposure alone do not guarantee adequate vitamin D status, particularly in vulnerable groups such as pregnant women.

Although previous reports from southern Europe have consistently documented high rates of maternal and neonatal hypovitaminosis D, most have relied primarily on prevalence estimates without integrating objective environmental exposures. More recent evidence underscores the importance of urban atmospheric factors and climate variability in shaping vitamin D status [39,40,41]. The present study advances this field by combining biochemical assessments with fine-scale environmental indicators such as UV index, sunshine duration, and PM2.5 concentrations. This integrative approach offers a novel perspective, illustrating how seasonal and pollution-related dynamics in an urban Mediterranean setting sustain insufficient vitamin D levels despite high solar availability. By linking maternal–neonatal outcomes with environmental parameters, our findings move beyond confirmation of deficiency prevalence and contribute to the emerging discourse on climate-sensitive maternal–fetal health. To our knowledge, few studies in southern Europe have explicitly integrated maternal–neonatal vitamin D status with high-resolution environmental metrics, highlighting the originality of the present work within the Mediterranean context [37,38].

Recent studies have further explored the environmental determinants of vitamin D status in pregnancy, showing negative associations between air pollution (especially PM2.5) and serum 25(OH)D levels in large cohorts [41,42]. These works, together with our findings, reinforce the importance of integrating urban atmospheric exposures into discussions of vitamin D deficiency in pregnant populations.

While the high prevalence of VDD has been reported in previous studies from Mediterranean as well as non-Mediterranean regions, the contribution of the present work lies in its combined assessment of biological outcomes with objective environmental exposures. By integrating serum measurements with data on UV index, sunshine duration, and air pollution, alongside maternal supplementation practices, this study moves beyond descriptive prevalence estimates. It highlights the specific climatic and atmospheric mechanisms that help sustain deficiency in an urban Mediterranean setting and demonstrates how these factors shape both maternal and neonatal vitamin D status.

Several mechanisms may underlie this disconnect. First, ambient UVB radiation is subject to seasonal attenuation due to changes in solar zenith angle, daylight duration, and atmospheric clarity, all of which are governed by climate-related seasonal cycles. Between October and March, UV index values averaged below 3, while PM2.5 levels exceeded 20 μg/m^3^ (Table 1), potentially limiting cutaneous vitamin D synthesis. Elevated PM2.5 levels may act synergistically with low solar angle and cloud cover to reduce UVB transmissibility, highlighting the complex interplay between climate, pollution, and health outcomes. Similar effects of air pollution on UVB transmissibility have been described in observational studies from urban settings [26,43]. Second, behavioral adaptation to the urban climate likely plays a role. In our analysis, sun exposure was significantly associated with 25(OH)D levels (*p* = 0.014), yet its effectiveness was modulated by season and pollution, indicating that self-reported exposure may not accurately reflect bioavailable UVB. Notably, sun avoidance, protective clothing, and indoor lifestyles during pregnancy are common in southern Europe [16], further reducing effective dermal synthesis. Such behavioral patterns can be seen as adaptive responses to the local urban climate, where high temperatures and intense sunlight paradoxically lead to sun avoidance and thus exacerbate vitamin D insufficiency. At the same time, limited dietary sources of vitamin D, such as oily fish or fortified dairy, are not systematically emphasized in maternal nutrition programs in Greece, which further restricts opportunities to achieve sufficiency during pregnancy. This underscores the value of integrating structured dietary assessment and tailored nutritional guidance into prenatal care visits, ensuring that food-based vitamin D sources and fortified options complement supplementation strategies. Integrating nutritional counseling into prenatal care frameworks could therefore serve as a complementary pathway to counterbalance reduced cutaneous synthesis.

A third factor is the limited efficacy of standard prenatal supplementation. Although supplemented mothers had higher serum levels (26.92 ± 12.43 ng/mL vs. 16.92 ± 9.57 ng/mL; Table 7), the majority remained insufficient, and 73% of their neonates were still deficient (Table 5). This may reflect suboptimal dosing, impaired placental transfer, or environmental interference. Seasonal analysis (Table 6) confirmed that winter supplementation was less effective, likely due to concurrent UV scarcity and high PM2.5. From a clinical perspective, this finding highlights that supplementation should not be treated as a stand-alone measure; embedding it within a broader prenatal nutrition framework may enhance effectiveness, particularly if aligned with dietary counseling and seasonal risk profiling.

These findings suggest that environmentally sensitive supplementation policies may be required, integrating climatic variables (season, UV index) and atmospheric pollutants (e.g., PM2.5) into public health recommendations. A fixed-dose supplementation model may be insufficient in the face of dynamic environmental stressors that alter dermal synthesis efficacy throughout the year.

The strong correlation observed between maternal and neonatal vitamin D levels (r = 0.800; Table 4, Figure 1) further supports the idea that shared environmental exposures shape dyadic vitamin D status, underscoring the importance of targeting maternal sufficiency as a strategy for improving neonatal health outcomes.

Importantly, multivariate analysis demonstrated that season and sun exposure remained independent predictors of maternal 25(OH)D, even when adjusting for smoking and parity (Table 9). These results emphasize the primacy of environmental variables—particularly UV radiation availability—in determining vitamin D status in pregnant populations, thereby reinforcing the need to view vitamin D status not solely as a nutritional or behavioral issue, but as a climate-modulated public health challenge.

These findings also point toward key methodological directions for future research. Longitudinal cohort studies integrating real-time climatic and atmospheric data—such as solar radiation levels, temperature variation, and air pollution indices—could offer deeper insight into the temporal dynamics of vitamin D status during pregnancy. The incorporation of wearable UV dosimeters in such cohorts would allow for objective, continuous measurement of individual UV exposure, addressing limitations of self-reported data and enhancing accuracy in exposure-outcome modeling. These approaches could inform the development of dynamic, environment-aware risk prediction tools and allow public health interventions to be tailored more precisely to the lived climatic realities of urban pregnant populations.

### 4.1. Strengths and Limitations

A major strength of this study is its integration of real-world environmental and climatic data (UV index, sunshine duration, temperature, PM2.5) with biochemical vitamin D measurements in a clinical population. To our knowledge, this is one of the few studies in southern Europe to combine environmental exposure profiling with maternal–neonatal vitamin D outcomes in a real urban setting.

However, the cross-sectional design precludes causal inference, as the associations observed cannot establish temporal or mechanistic relationships. Although our analyses included regression modeling and correlation testing to move beyond descriptive patterns, the scope of statistical inference remains constrained by the study design. Additionally, the restricted inclusion criteria, focusing on women with long-term residency in Greece and uncomplicated term singleton pregnancies, may limit the generalizability of our findings. Nevertheless, this approach enhanced internal validity by ensuring homogeneity and minimizing potential confounding. The timing of blood sampling (day of delivery) may not reflect long-term status, and sun exposure was self-reported, introducing recall bias. We also lacked data on dietary intake, genetic polymorphisms, or vitamin D-binding protein levels, which could influence interindividual variability. The absence of detailed dietary data is particularly relevant in a prenatal context, where maternal nutritional practices directly influence fetal development and should ideally be considered alongside environmental exposures. Future work should therefore integrate validated dietary assessment tools into maternal monitoring protocols, as diet represents a modifiable determinant that can be directly addressed within prenatal care.

### 4.2. Public Health Implications

These findings have important public health implications. From a nutritional standpoint, prenatal care should emphasize both supplementation and dietary counseling, ensuring that pregnant women are aware of natural and fortified food sources of vitamin D. Incorporating this counseling systematically into routine antenatal visits, similar to folate and iron guidance, would ensure that vitamin D is addressed consistently within the broader framework of prenatal nutrition. This dual approach may provide greater resilience against environmentally constrained dermal synthesis. Vitamin D guidelines for pregnant women may need seasonally adjusted and pollution-aware dosing strategies, particularly in urban regions where air quality attenuates UV effectiveness. Routine screening and dynamic adjustment of supplementation protocols may help prevent deficiency-related outcomes in both mothers and neonates.

Future research should explore longitudinal cohort designs incorporating wearable UV dosimeters, dietary assessments, and direct pollution exposure mapping. Environmental health should be viewed as a critical dimension of maternal–fetal medicine in the era of urbanization and climate change.

Moreover, climate-informed health strategies should consider the integration of UV forecast data, seasonal pollution levels, and urban climate parameters into prenatal care frameworks. The incorporation of climate–health linkages into maternal health policy may enhance resilience to environmental variability in urban populations.

The study has certain limitations. The sample was context-specific, restricted to urban, term singleton pregnancies among long-term residents in Greece. Some variables were based on self-report, even though information was cross-checked against medical records. Despite adjustment for multiple covariates, the possibility of residual confounding cannot be excluded. These considerations should be taken into account when interpreting the results. Nevertheless, the findings are likely generalizable to urban Mediterranean settings with comparable climatic and air quality profiles.

## 5. Conclusions

Despite abundant solar radiation in the Mediterranean region, this study identified a persistently high burden of VDD among pregnant women and neonates in an urban Mediterranean climate. The findings underscore the interplay of seasonal UV availability, air pollution, behavioral adaptations, and maternal diet, highlighting the need for prenatal care models that integrate environmental awareness with tailored nutritional strategies. The observed inadequacy of standard prenatal supplementation, particularly during low-sunlight months, illustrates the limitations of uniform dosing strategies and indicates that seasonally adjusted protocols may warrant consideration. These results point to the potential value of integrating environmental exposure metrics such as UV indices and air pollution data into maternal health policy and vitamin D guidelines, in order to inform context-specific strategies. Future research should adopt longitudinal designs with real-time environmental monitoring and wearable technologies to better capture the dynamic relationship between atmospheric conditions, behaviors, and vitamin D status during pregnancy.

These findings may inform prenatal care policies under seasonal and air quality constraints, highlighting the integrated linkage of maternal–neonatal vitamin D status with environmental exposures.

## Figures and Tables

**Table 1:** Indicative Environmental Variables (UV, PM2.5, Temperature) During Key Months of the Study Period.

Month	UV Index (avg)	PM2.5 (μg/m^3^)	Sunshine Hours (avg/Day)	Mean Temp (°C)
January	2.1	27.3	3.1	10.2
March	4.8	18.2	5.9	13.9
May	7.6	12.7	9.2	21.6
July	9.5	9.4	11.3	28.1
September	7.8	11.6	9.4	24
November	3.2	20.1	4.1	15.4

Data retrieved from NASA POWER and National Observatory of Athens archives. Values represent monthly averages; no statistical test applied.

**Table 2:** Mean Maternal and Neonatal 25(OH)D Levels by Season (Groups A and B).

Group	Period	Maternal 25(OH)D (ng/mL)	Neonatal 25(OH)D (ng/mL)
A	April–October (Warm season)	24.22 ± 12.57	16.37 ± 8.55
B	October–March (Cold season)	16.96 ± 9.60	12.87 ± 8.20

Group comparisons performed using independent samples *t*-test for normally distributed maternal 25(OH)D values and Mann–Whitney U test for skewed neonatal 25(OH)D values.

**Table 3:** Prevalence of vitamin D deficiency, insufficiency, and sufficiency in mothers and neonates per season. Cutoffs based on Endocrine Society guidelines.

Group	Deficient (<20 ng/mL)	Insufficient (20–29 ng/mL)	Sufficient (≥30 ng/mL)
Mothers—Group A	45 (36.9%)	47 (38.5%)	30 (24.6%)
Mothers—Group B	92 (67.6%)	29 (21.3%)	15 (11.0%)
Neonates—Group A	91 (74.6%)	20 (16.4%)	11 (9.0%)
Neonates—Group B	124 (89.9%)	11 (8.0%)	3 (2.2%)

Differences between groups assessed using Chi-square test.

**Table 4:** Pearson Correlation between Maternal and Neonatal 25(OH)D Levels.

Correlation Pair	r	*p*-Value
Maternal vs. Neonatal 25(OH)D	0.8	<0.001

Pearson correlation coefficient (r) with two-tailed significance.

**Table 5:** Neonatal Vitamin D Status According to Maternal Supplementation and Potential Environmental Interference.

Maternal Supplementation	Neonatal Mean 25(OH)D (ng/mL)	% Neonates < 20 ng/mL
Yes (*n* = 83)	15.3 ± 8.1	73%
No (*n* = 165)	13.2 ± 8.6	88%

Differences in neonatal mean 25(OH)D assessed using Mann–Whitney U test due to skewed distribution; categorical comparisons assessed using Chi-square test.

**Table 6:** Neonatal Vitamin D Deficiency by Season and Maternal Supplementation Status.

Group	Supplemented Mothers	Neonatal Deficiency Rate (<20 ng/mL)
Warm season (A)	Yes (*n* = 50)	66%
Cold season (B)	Yes (*n* = 33)	82%

Differences between groups assessed using Chi-square test.

**Table 7:** Maternal 25(OH)D Status Based on Prenatal Vitamin D Supplementation.

Maternal Supplementation	Mean Maternal 25(OH)D (ng/mL)	% Deficient (<20 ng/mL)	% Insufficient (20–29 ng/mL)	% Sufficient (≥30 ng/mL)
Yes (*n* = 83)	26.92 ± 12.43	32%	30%	38%
No (*n* = 165)	16.92 ± 9.57	64%	25%	11%

Maternal mean 25(OH)D compared using independent samples *t*-test; categorical comparisons of deficiency categories assessed using Chi-square test.

**Table 8:** Univariate Associations Between Maternal 25(OH)D and Demographic or Behavioral Variables.

Variable	*p*-Value	Significance
Season (Group A vs. B)	<0.001	Significant
Sun exposure	0.014	Significant
Smoking status	0.002	Significant
Parity	0.004	Significant
BMI	0.722	** NS
Socioeconomic status	0.202	** NS
Physical activity	0.508	** NS

Not retained in further analysis due to lack of statistical and environmental relevance. Continuous variables tested with independent samples *t*-test or Mann–Whitney U test depending on distribution; categorical variables tested with Chi-square test. Abbreviations: ** NS = Not significant.

**Table 9:** Multivariate Linear Regression Model for Maternal 25(OH)D Levels.

Predictor Variable	Standardized β	*p*-Value	Adjusted R^2^
Season (warm vs. cold)	0.42 (0.28, 0.56)	<0.001	0.28
Sun exposure (h/day)	0.26 (0.06, 0.46)	0.011	
Smoking (yes vs. no)	−0.15 (−0.30, −0.01)	0.048	
Parity (≥1 vs. nullip.)	0.09 (−0.03, 0.22)	0.12	

Results derived from multivariate linear regression model with maternal 25(OH)D as the dependent variable. Note: Standardized β coefficients are reported with 95% confidence intervals. Adjusted R^2^ for the overall model was 0.28.

## Data Availability

The original contributions presented in this study are included in the article. Further inquiries can be directed to the corresponding author.

## Supplementary Materials

The following supporting information can be downloaded at: https://www.mdpi.com/article/10.3390/healthcare13202568/s1, Supplementary Table S1: STROBE Statement Checklist. Supplementary Table S2: Full Multivariable Regression Outputs. Supplementary file: Supplementary Methods.
