# Supplementary material for: Seasonal and Environmental Determinants of Maternal and Neonatal Vitamin D Status: A Cross-Sectional Observational Cohort Study in Urban Greece
Source: Healthcare (Basel). 2025 Oct 13;13(20):2568. doi: 10.3390/healthcare13202568 (PMC12563419; doi:10.3390/healthcare13202568)
Supplement: Supplementary file 1 [file healthcare-13-02568-s001.zip › Supplementary file Supplementary Methods.pdf]

Supplementary Methods

This Supplementary Methods file provides detailed information on the data sources used for environmental and climatic variables in the study.

Environmental Data Sources

- NASA POWER database: https://power.larc.nasa.gov/

- National Observatory of Athens (NOA): https://www.noa.gr/

- Hellenic National Meteorological Service (HNMS/EMY): http://www.hnms.gr/

- Ministry of Environment and Energy (Ypen): https://ypen.gr/

- OpenAQ platform: https://openaq.org/

These URLs provide access to the raw environmental datasets used in the analyses, including UV index, solar radiation, sunshine duration, temperature, rainfall, and air pollution indicators (PM2.5, NO_2, O_3).
