# Supplementary material for: Seasonal and Environmental Determinants of Maternal and Neonatal Vitamin D Status: A Cross-Sectional Observational Cohort Study in Urban Greece
Source: Healthcare (Basel). 2025 Oct 13;13(20):2568. doi: 10.3390/healthcare13202568 (PMC12563419; doi:10.3390/healthcare13202568)
Supplement: Supplementary file 1 [file healthcare-13-02568-s001.zip › Supplementary Table S1 STROBE Statement Checklist.pdf]

Supplementary Table S1: STROBE Statement Checklist

Manuscript title: Seasonal and Environmental Determinants of Maternal and Neonatal Vitamin D Status: A Cross-Sectional Observational Cohort Study in Urban Greece

Authors: Kokkinari A, Dagla M, Gourounti K, Sarantaki A, Kirkou G, Iliadou M, Antoniou E, Iatrakis G

Item	STROBE Recommendation	Manuscript Location/Description
Title and Abstract	Indicate study design in the title or abstract; provide informative summary of what was done and found.	Title specifies cross-sectional observational cohort; abstract structured with background, methods, results, conclusions.
Introduction: Background/Rationale	Explain scientific background and rationale for investigation.	Introduction describes Mediterranean paradox, vitamin D deficiency, environmental exposures, and rationale for study.
Introduction: Objectives	State specific objectives and hypotheses.	Objective to investigate maternal–neonatal vitamin D status in relation to seasonal and environmental determinants.
Methods: Study Design	Present key elements of study design early.	Cross-sectional observational cohort with linked environmental exposures, described in Methods 2.1.

Methods: Setting	Describe setting, locations, and dates.	Urban hospital in Piraeus, Greece; data collected between September 2019 and January 2022.
Methods: Participants	Give eligibility criteria, sources, selection methods.	Singleton term pregnancies, ≥37 weeks, exclusions for chronic disease/medications. Flow paragraph added: 312 approached, 64 excluded, 248 included.
Methods: Variables	Clearly define outcomes, exposures, predictors, potential confounders.	Primary outcome: maternal and neonatal 25(OH)D. Exposures: UV index, sunshine, temperature, PM2.5. Confounders: age, BMI, smoking, parity, supplementation.
Methods: Data sources/measurement	For each variable, give data sources and details of measurement.	Vitamin D measured by CMIA assay. Environmental data from NOA, HNMS, NASA POWER, Ministry/OpenAQ. Monthly means aligned to gestational months.
Methods: Bias	Describe efforts to address potential sources of bias.	Cross-checking of self-reported data with medical records; exclusion of high-risk pregnancies to reduce confounding.
Methods: Study size	Explain how study size was arrived at.	Final analytic cohort of 248 term singleton pregnancies

		after exclusion of ineligible participants.
Methods: Quantitative variables	Explain handling of quantitative variables.	Vitamin D categorized per Endocrine Society cutoffs; seasonal grouping defined by HNMS warm/cold periods.
Methods: Statistical methods	Describe methods, including confounder control.	Descriptive statistics, parametric/non-parametric tests, univariate and multivariate regression with β, OR, 95% CI, adjusted R^2 reported.
Results: Participants	Report numbers at each stage and reasons for non-participation.	312 approached, 64 excluded, final N=248. Flow description provided in Methods 2.1.
Results: Descriptive data	Provide characteristics of study participants.	Maternal demographics, supplementation, neonatal characteristics detailed in Tables 1–7.
Results: Outcome data	Report numbers of outcome events or summary measures.	Maternal and neonatal 25(OH)D levels reported by season, supplementation, correlation (Tables 2–7).
Results: Main results	Give unadjusted and adjusted estimates, precision, and report confounder control.	Univariate and multivariate regression models provided with β, OR, CI; adjusted R^2 presented.
Results: Other analyses	Report subgroup and sensitivity analyses.	Seasonal stratification, supplementation

		subgroups, environmental interactions analyzed.
Discussion: Key results	Summarise key results with reference to objectives.	Persistent maternal–neonatal insufficiency despite high solar availability; environmental exposures influence status.
Discussion: Limitations	Discuss study limitations, direction and magnitude of potential bias.	Cross-sectional design; context-specific sample; self-report; lack of dietary/genetic data; residual confounding.
Discussion: Interpretation	Give cautious overall interpretation, considering objectives, limitations, other studies.	Findings interpreted within Mediterranean paradox literature, highlighting environmental determinants and public health relevance.
Discussion: Generalisability	Discuss external validity of results.	Findings generalisable to urban Mediterranean settings with similar climate and air quality profiles.
Other: Funding	Give source of funding and role of funders.	No external funding; stated in manuscript.
