# Supplementary material for: Seasonal and Environmental Determinants of Maternal and Neonatal Vitamin D Status: A Cross-Sectional Observational Cohort Study in Urban Greece
Source: Healthcare (Basel). 2025 Oct 13;13(20):2568. doi: 10.3390/healthcare13202568 (PMC12563419; doi:10.3390/healthcare13202568)
Supplement: Supplementary file 1 [file healthcare-13-02568-s001.zip › Supplementary Table S2 Full Multivariable Regression Outputs.pdf]

Supplementary Table S2. Full Multivariable Regression Outputs

The following table presents the complete results from the multivariable linear regression model with maternal serum 25(OH)D concentration as the dependent variable.

Predictor Variable	Standardized β	95% CI	p-value	Adjusted R^2
Season (warm vs cold)	0.42	0.28 – 0.56	<0.001	0.28
Sun exposure (hrs/day)	0.26	0.06 – 0.46	0.011	
Smoking (yes vs no)	-0.15	-0.30 – -0.01	0.048	
Parity (≥1 vs nullip.)	0.09	-0.03 – 0.22	0.120	

Note: Standardized β coefficients are reported with 95% confidence intervals. Adjusted R^2 for the overall model was 0.28.
